# Examining memory reconsolidation as a mechanism of nitrous oxide’s antidepressant action

**DOI:** 10.1038/s41386-024-02049-0

**Published:** 2025-01-17

**Authors:** Ella Williams, Ursule Taujanskaite, Sunjeev K. Kamboj, Susannah E. Murphy, Catherine J. Harmer

**Affiliations:** 1 Department of Psychiatry, Oxford University, Warneford Hospital, Oxford, UK; 2 Clinical Psychopharmacology Unit, Research Department for Clinical, Educational and Health Psychology, University College London, 1-19 Torrington Place, London, UK; 3 Oxford Health NHS Foundation Trust, Warneford Hospital, Oxford, UK

**Keywords:** Translational research, Long-term memory, Human behaviour

## Abstract

There is an ongoing need to identify novel pharmacological agents for the effective treatment of depression. One emerging candidate, which has demonstrated rapid-acting antidepressant effects in treatment-resistant groups, is nitrous oxide (N_2_O)—a gas commonly used for sedation and pain management in clinical settings and with a range of pharmacological effects, including antagonism of NMDA glutamate receptors. A growing body of evidence suggests that subanaesthetic doses of N_2_O (50%) can interfere with the reconsolidation of maladaptive memories in healthy participants and across a range of disorders. Negative biases in memory play a key role in the onset, maintenance, and recurrence of depressive episodes, and the disruption of affective memory reconsolidation is one plausible mechanism through which N_2_O exerts its therapeutic effects. Understanding N_2_O’s mechanisms of action may facilitate future treatment development in depression. In this narrative review, we introduce the evidence supporting an antidepressant profile of N_2_O and evaluate its clinical use compared to other treatments. With a focus on the specific memory processes that are thought to be disrupted in depression, we consider the effects of N_2_O on memory reconsolidation and propose a memory-based mechanism of N_2_O antidepressant action.

## Introduction

For many years, modulations in the neurotransmitter, serotonin, have been posited to play a pivotal role in the aetiology and treatment of depression [1–3]. Indeed, the most common first-line antidepressant medications (e.g., selective serotonin reuptake inhibitors (SSRIs)) act via the potentiation of serotonin neurotransmission [4, 5]. However, around a third of individuals with depression do not respond to these conventional treatments [6, 7], and meet diagnostic criteria for ‘treatment-resistant depression’ (TRD). Furthermore, for individuals who *do* respond to these monoamine-based treatments, the remediation and alleviation of symptoms can take many weeks [8]. Therefore, there is an outstanding clinical need to identify novel pharmacological agents for the effective treatment of depression.

The N-methyl-D-aspartate glutamate receptor (NMDAR) has emerged as an alternative antidepressant target, with increasing evidence suggesting that pharmacologically blocking or antagonising this receptor leads to rapid-acting antidepressant effects for individuals with TRD [9]. At present, the most notable NMDAR antagonist for depression treatment is ketamine [10]. Whilst s-ketamine (or esketamine) has been developed and approved as an antidepressant nasal spray in the United States [11] and some areas of Europe [12], there are a number of challenges and concerns associated with its use. For example, ketamine is a controlled drug in the UK, and is under similarly strict regulatory controls in many other countries [13]. Moreover, ketamine use is linked with significant physiological and psychological side effects (e.g., tachycardia, raised blood pressure, nausea, headaches, disorientation, anxiety) [14, 15]. Beyond the acute side effects of ketamine, its long-term use has additionally been linked with lasting urinary and gastrointestinal damage [16]. In light of these issues, other antidepressant NMDAR antagonists with enhanced safety profiles are continuing to be considered [17, 18].

One suggested alternative to ketamine is nitrous oxide (N_2_O). Like ketamine, N_2_O is a dissociative anaesthetic, which has been used in dental and general surgery since the mid-nineteenth century. It also serves as a common method of conscious sedation and analgesia [19–21]. More recently, N_2_O has shown promise as a treatment for a number of psychiatric conditions, including post-traumatic stress disorder (PTSD) [22]. Most pertinent to the current review, however, is the demonstration that N_2_O exhibits rapid-acting, yet somewhat transient, antidepressant effects in individuals with TRD [18, 23]. A randomised control trial recorded antidepressant effects which were present 2 and 24 h after a single N_2_O inhalation session, but not 1 week post-inhalation [24]. There are some studies that have suggested longer-lasting effects; one case report stated that total remission from depression was achieved for a patient following an N_2_O inhalation session, and this effect lasted at least one month [25]. The standard procedure for treatment, which was used for the previously mentioned studies, involves a single 60-minute inhalation session using 50% nitrous oxide. However, effects of N_2_O in TRD lasting two weeks have also been reported with both 25% and 50% nitrous oxide [23].

Investigations employing multiple inhalation sessions have additionally been conducted; in a double-blind placebo-controlled trial, Guimarães and colleagues recorded acute and cumulative reductions in depressive symptoms following multiple inhalation sessions (60-minutes with 50% N_2_O), which occurred twice a week for four weeks [26]. However, no follow-up results were reported, meaning the longevity of N_2_O’s antidepressant effect following multiple inhalation sessions is as yet unknown. In addition to the published trials assessing N_2_O’s effects in depression (see Table 1), 13 others have also been registered, but have not yet reported results (six complete, one active but not recruiting, four recruiting and two not yet recruiting). See also reviews [27, 28].

**Table 1:** Summary of published findings.

Study	*n*	Condition	N_2_O Dose	Control	No. of Sessions	Primary Outcome	Timepoints	% Response in N_2_O Group	% Remission in N_2_O Group	Group Comparison (N_2_O vs placebo)
Guimarães et al. 2021	**21**^**a**^ *N*_*2*_*O* = *12* *placebo* = *9*	MDD	50%	100% oxygen	8	HAM-D17	Before and after each session (twice a week for four weeks)	91.7	75	*Time-drug interaction*^*c*^ *p* < 0.005
Nagele et al. 2015	**20**^**b**^ *crossover trial*	TRD	50%	50% nitrogen: 50% oxygen	1	HDRS-21	2 h 24 h	20	15	*p* < 0.001
Nagele et al. 2020	**1**	MDD	50%	n/a	1	PHQ-9	1 month	n/a	100	n/a
Nagele et al. 2021	**24**^**b**^ *crossover trial*	TRD	25% and 50%	air/oxygen	2	HDRS-21	2 h 24 h 1 week 2 weeks	41.7 (50%), 33.3 (25%)^d^	41.7 (50%), 22.2 (25%)^d^	*p* = 0.01
Yan et al. 2022	**42**^**b**^ *N*_*2*_*O* = *20* *placebo* = *22*	TRD	50%	50% air: 50% oxygen	1	HDRS-21	2 h 24 h 1 week 2 weeks	10 (24 h) 10 (1 week) 30 (2 weeks)	0 (24 h) 10 (1 week) 15 (2 weeks)	*p* = 0.017 (2 h) *p* = 0.033 (24 h) *p* = *0.463 (1 week)* *p* = *0.711 (2 weeks)*

The bold text shows the sample size and the italics provide additional information about the group allocations.

*MDD* major depressive disorder, *TRD* treatment-resistant depression, *HAM-D17* 17-item Hamilton Depression Rating Scale, *HDRS-21* 21-item Hamilton Depression Rating Scale, *PHQ-9* Patient Health Questionnaire.

^a^n based on total number of participants who completed the trial.

^b^n based on modified intention-to-treat analyses.

^c^Significant group differences observed post-session 4 (*p* = 0.027), post-session 7 (*p* = 0.008), pre-session 8 (*p* = 0.046) and post-session 8 (*p* = 0.003).

^d^This analysis only included data where pre-treatment HDRS-21 scores were ≥19.

N_2_O is believed, like ketamine, to act primarily as a non-competitive NMDAR antagonist [17, 29–31], although interactions with other receptors have also been noted. Beyond its anaesthetic actions, which are attributed to its NMDAR antagonism, N_2_O also has antinociceptive and anxiolytic properties [32]. Whilst the precise mechanism of N_2_O’s action at these receptors is currently unclear, the pain modulatory effects of N_2_O have been associated with its likely indirect stimulation of opioid receptors [33, 34], while the anxiolytic effects have been linked to activation of GABA_A_ receptors via the benzodiazepine binding site [35, 36]. Ketamine and N_2_O have similar effects on neuronal behaviour, and both generate plasticity in neuronal circuits through overlapping, though not identical, biochemical pathways [37]. A notable distinction between ketamine and N_2_O relates to the speed at which they are eliminated from the body. Ketamine remains pharmacologically active for a number of hours and its clinical effects may be partly mediated by its metabolites [17]. By contrast, N_2_O has no active metabolites and is rapidly eliminated through the lungs due to its low solubility in blood [38, 39]. Despite these differences, the antidepressant effects of both agents appear to be sustained long after their elimination [17].

At the psychological level, N_2_O has been shown to induce both dissociative and psychotomimetic symptoms to an extent comparable to ketamine [40]. When compared with ketamine, however, N_2_O use is accompanied by a lower risk of adverse side effects [41], and its abuse and addiction potential is considered to be significantly lower [17]. Some of the side effects associated with N_2_O use are nausea, vomiting and occasional paradoxical increases in anxiety. However, adverse events are uncommon (4.4%), minor, and resolve rapidly following inhalation cessation [17, 39, 41]. Accordingly, N_2_O is thought to be an exceptionally safe drug [42]. As a consequence, it is not as strictly regulated as many other narcotic or dissociative agents, making N_2_O a relatively accessible compound to investigate as a potential treatment for depression.

The specific mechanisms underpinning the effects of N_2_O in depression are yet to be fully elucidated. The current review will focus on the proposition that N_2_O’s antidepressant action could be linked to its ability to interfere with the reconsolidation of maladaptive memories. We first summarise the affective memory biases that are typically seen in depression and review the evidence base for memory therapeutics in order to outline the rationale for a treatment which might target memory processing (and specifically memory reconsolidation). We then introduce some molecular, cellular and behavioural mechanisms of memory reconsolidation and evaluate evidence for a memory-based mechanism of N_2_O’s drug action. Finally, we highlight some of the open questions remaining in this field, potential challenges associated with the implementation of N_2_O-based therapies, and possible directions for future research.

## Memory in depression

### Explicit and implicit affective memory biases

A large body of research indicates that individuals with depression tend to remember more negative information than healthy controls (who typically show a positive memory bias) [43–50]. This negative memory bias in depression has been demonstrated using a variety of stimuli, including affective pictures [45], self-referential personality trait words [46, 51], emotional stories [52, 53], and facial expression stimuli [47, 54]. This bias has also been reported across multiple tests of explicit memory, but has tended to be more consistently observed in free recall over recognition tasks [55].

Beyond these measures of explicit memory, in which information is consciously and effortfully stored and retrieved, individuals with depression also demonstrate negative biases in implicit memory, which rather involves the storage and retrieval of information outside of conscious awareness [55, 56]. For measures of emotionally biased implicit memory, participants are first exposed to a list of affectively valenced words (e.g., through word categorisation tasks) without any instructions to learn the items. Then, at a later stage, participants complete an ostensibly unrelated task (e.g., word stem completion, free word association). It has been reported that, compared with healthy controls, individuals with depression tend to respond to these secondary tasks with a higher proportion of negatively valenced words from the primed lists as opposed to primed positive, neutral, or novel words. This is interpreted as an implicit negative memory bias; the negative information appears to have been preferentially encoded and retrieved without effort or deliberation [43, 48–50]. Cognitive theories of depression suggest that that these memory biases are closely linked with the development, maintenance, and relapse of depression [57–60] and that they arise through the increased elaboration of negative information. In other words, negative information is integrated more robustly into pre-established memory networks through the connection of the negative memory representation with other semantic associations [61, 62].

### Autobiographical memory in depression

#### Overgeneral autobiographical memory

Reflecting on personal memories provides a scaffold for one’s self-perception and identity [63]. In depression, there is consistent evidence that the recall of such autobiographical memories is disrupted. One of the most robust findings is that depression is associated with overgeneral autobiographical memory recall [64, 65]. That is to say, individuals with depression often remember autobiographical memories with reduced specificity. Cognitive neuropsychological theories posit that adverse life experiences bestow a particular susceptibility to depression through the formation and consolidation of negative categorical themes, or *schemas*, which shape the way in which individuals habitually interpret and interact with their environment [66–68]. It is therefore possible that these schemas contribute to the overgeneral autobiographical memory phenomenon as these abstract representations impede the retrieval of specific autobiographical information [69, 70]. Other related explanations for the overgeneral autobiographical memory phenomenon in depression include i) the presence of ruminative thinking patterns, which compete for the cognitive resources required for the retrieval of specific details about the past, ii) functional avoidance as the encoding and retrieval of specific memories threatens to hijack attention and cause affective disturbances, and iii) deficits in executive control [70, 71]. Investigations into whether this effect is seen specifically for negative memories in depression have yielded inconsistent findings [64, 72]. However, a recent meta-analysis suggested that it exists irrespective of the emotional valence of the memory cue presented [65].

#### Negatively biased autobiographical memory

Affective biases in the recall of autobiographical memory have also been reported in depression. For example, negative memories are spontaneously recalled more than positive ones, and individuals with depression are quicker to retrieve negative memories in tasks requiring the recall of life events [73]. These emotionally valenced patterns of recall may be underpinned by the negative attentional and interpretation biases commonly reported in depression, as these biases could lead to the preferential encoding of negative autobiographical information [44, 57, 74–76]. It is also likely that this enhanced access to negative memories contributes to the negative biases seen in the content and processing of thoughts that individuals with depression have about themselves, the world and the future—collectively termed the cognitive triad [66, 77]. Cognitive models further posit that the enhanced elaboration of negative memories may reinforce maladaptive emotion regulation strategies, such as rumination, which, for individuals with depression, often involves the ‘recycling’ of thoughts linked to the causes and consequences of their depressed mood [78–82].

In addition, negative autobiographical memories are commonly intrusive in nature as they are recalled spontaneously and involuntarily in depressed individuals [73, 79, 83–85]. Sometimes these intrusive memories lead people to feel as though they are emotionally and/or physically re-living the events, which can be particularly distressing [86–88]. The content of intrusive memories frequently relates to life events which reinforce the negative views that depressed individuals hold about themselves and their future [86]. Given the emotional power of this reexperiencing and the negative associations attached to the memories, it is unsurprising that a relationship has been identified between intrusion levels and the severity of an individual’s depressive symptoms [84, 89].

In contrast, positive autobiographical memories are often less vivid [90], recalled less frequently [91, 92], and recollected more slowly [93] than negative or neutral memories in people with depression. This has significant implications for the regulation of mood; the retrieval of positive autobiographical memories has been shown to have a positive impact on subjective mood in healthy volunteers. However, this effect is much reduced, or even reversed, in individuals with a history of depression [94, 95].

## Memory therapeutics: counteracting the influence of maladaptive memories in psychopathology

Maladaptive memory is common to many psychopathologies in addition to depression (e.g., PTSD, OCD, alcohol or drug addiction). Accordingly, efforts to counteract the influence of such memories could prove to be particularly beneficial for transdiagnostic symptom suppression and/or treatment. Memory representations are not static entities; they go through a series of intricate and dynamic processing stages, which could be targeted for clinical benefit. For example, strategies could be employed during the initial memory ***encoding*** or acquisition phase, so that the original formation of the maladaptive memory is interrupted (i.e., primary prevention) [96, 97]. However, whilst it is possible to administer interventions prospectively in an experimental set-up, targeting memory encoding in a therapeutic context is practically challenging due to the unpredictability of maladaptive memory-forming events. Following these initial stages of encoding, memories undergo a stabilisation process (i.e., memory ***consolidation***) for several hours before being transferred into long-term storage. Targeting this consolidation period could therefore offer a slightly extended, though still time-limited, window of opportunity for therapeutic memory interference [98, 99].

According to the dominant consolidation-based account of long-term memory [100], once encoded and consolidated into long-term storage, memories become impervious to amnestic agents and their contents and strength (i.e., ease of retrieval) do not change substantially over short periods of time. However, it has long been recognised that the expression of long-term memories can nonetheless be modified using behavioural extinction procedures [101, 102]. One explanation for this is that these behavioural techniques involve the formation of alternative memories that compete with existing memories during ***retrieval***. In a similar way, cognitive therapies may be used to generate and bolster competing memory representations. An important aspect of these extinction-based interventions is that they do not target the original memory, but instead aim to create alternative representations which interfere with the original memory trace and reduce the likelihood of its retrieval [103]. Given that the original memory remains intact, it retains its capacity to re-exert control over behaviour. In the context of psychopathologies, the re-emergence of symptoms after successful treatment (i.e., relapse) is possible through several mechanisms which cause the original memory trace to become dominant once more (e.g., renewal, spontaneous recovery or reacquisition) [104].

In contrast to this inhibitory retrieval perspective [105], ***reconsolidation*** theory proposes a process whereby a previously consolidated memory trace is made labile again through its reactivation (or ‘destabilisation’) and thus becomes temporarily susceptible to interference before it undergoes another consolidation-like process (hence *re*consolidation). As such, the window of opportunity for intervention is significantly protracted as the reactivation process can take place long after the original consolidation of the memory. During reconsolidation, memories can be restabilised in updated forms (strengthened, weakened or qualitatively modified [106]), meaning that the risk of unwanted spontaneous recovery ought to be eradicated as the original maladaptive memory trace should not persist. The notion that the retrieval of an established (long-term) memory can induce such memory plasticity can be traced back to the seminal work of Bartlett nearly a century ago [107]. The first compelling demonstration of this retrieval-dependent amnesia was offered in 1968 [108], after which a sporadic interest amongst researchers emerged only in the late 1990s [109–111]. Since then, the molecular, cellular and behavioural bases for this phenomenon have been the focus of intense research, especially because its clinical application has been recognised as potentially transformational for disorders of maladaptive memory [112].

## Molecular, cellular, and behavioural mechanisms of memory reconsolidation

Like consolidation, reconsolidation is crucially dependent upon glutamate signalling. Although beta-adrenergic activity is also implicated, at least some types of memory are especially sensitive to glutamatergic manipulations [113]. In particular, N-methyl-d-aspartate (NMDA) glutamate receptor activity, its downstream targets and resultant protein synthesis, have been highlighted as critical components of memory reconsolidation [109, 114–116]. While memory *retrieval* requires GluA1-containing AMPA receptor (AMPAR) activity [117], the *destabilisation* and *restabilisation* of memories are dependent upon NMDARs containing the GluN2B unit and the GluN2A unit, respectively [118]. The downstream molecular cascades activated by NMDARs culminate in enhanced translation of plasticity-related proteins involved in memory maintenance [115, 119].

Preclinical experimental studies in rodents support these proposed neural and molecular mechanisms of memory reconsolidation; NMDAR antagonists have been shown to interfere with the reconsolidation of spatial memories [120], fear memories [121], and appetitive drug memories [122, 123]. These findings suggest that NMDAR blockade during the restabilisation phase of reconsolidation could – via indirect inhibition of synaptic protein synthesis – impair memory maintenance. In theory, a pharmacological agent like N_2_O could therefore have similarly disruptive effects on these memory processes through its antagonism at NMDARs. Indeed, N_2_O may be a particularly appropriate candidate as it has fast onset and offset dynamics. That is to say, given the divergent mechanisms involved in the different phases of memory reconsolidation (e.g., retrieval, destabilisation and restabilisation), the pharmacokinetics of N_2_O’s action may be especially useful for targeting specific temporal components of this memory-maintenance process [124].

However, it is also important to consider the challenges associated with extrapolating effects from preclinical animal work to humans. For example, in the human studies reviewed in this paper, both N_2_O and ketamine are administered at subanaesthetic doses, which likely block < 50% of native NMDA receptors [31]. In comparison, preclinical studies which assess memory (re)consolidation effects using agents such as MK-801 can achieve much greater levels of antagonism with fewer concerns about toxicity. Similarly, genetic down-regulation strategies that completely silence NMDA receptor signalling also interfere with memory reconsolidation in rodents [120, 121]. If higher levels of antagonism of NMDARs are required to generate more robust memory re-writing effects in humans, this may present significant translational challenges. In a recent study of reward (alcohol) memory rewriting in heavy drinkers [125], ketamine’s effects on clinical outcomes (alcohol consumption and craving) were predicted by its post-infusion plasma levels. This suggests that higher doses might lead to more powerful reconsolidation-modulation and more pronounced symptomatic improvement. Whilst some effect on memory reconsolidation appears to persist even with the subanaesthetic doses of NMDAR antagonists that are more commonly used in human experimental models, it is important to note that considerably higher concentrations of ketamine were used for this reward memory study. As such, if still higher doses are required for optimal responding, this implies the need for significant resources (potentially including those required for complete anaesthesia) for the effective clinical implementation of these therapeutic strategies.

Finally, the conditions under which a memory is retrieved appear to have implications for its reactivation and reconsolidation. Specifically, in order for a memory to be successfully ‘updated’, the occurrence of a relevant prediction error (where expectations and outcomes are mismatched) may be critical for its reactivation or destabilisation [126]. There are a number of ways that this expectancy violation is currently being implemented in reconsolidation experiments in humans (e.g., a surprise interruption during memory recall, or the omission of an expected reinforcer following memory retrieval) [124]. Whether and how these experimental prediction error manipulations can be translated to clinical settings remains to be determined.

## Nitrous oxide and reconsolidation of maladaptive memories

Although observations of the effects of N_2_O on human memory date back several decades [127–129], work has only recently started to assess its effects on memory consolidation and reconsolidation. In a human experimental study, a 30 min inhalation session of 50% N_2_O following a trauma film paradigm increased the rate of intrusive memory reduction during the following week, compared to a control condition (medical air) [130]. Since N_2_O was administered immediately after the initial encoding of the traumatic memory, this finding suggests an effect on early (synaptic) consolidation, which may be linked to the inhibiting effects that NMDAR antagonism can have on downstream plasticity-related protein synthesis during early long-term potentiation (LTP).

Extending these findings to memory *re*consolidation, it has been found that N_2_O administration, following alcohol memory re-activation, reduced subsequent drinking, as well as alcohol-cue-induced urge to drink in a group of hazardous drinkers [124]. These effects are suggested to be a result of N_2_O-induced interference with alcohol-related memory reconsolidation. Importantly, the retrieval of the alcohol memory was associated with a prediction error, generated via an unexpected reward omission. The main finding from this study derives from post-hoc analyses which indicated that the experienced level of prediction error, which was indirectly assessed through subjective ratings of surprise following the omission of the expected alcohol reinforcer, determined the extent to which N_2_O interfered with the reconsolidation of the alcohol memory (reflected in a reduction in alcohol consumption) [124]. These findings therefore suggest that, under optimised retrieval conditions, N_2_O may offer an effective reconsolidation-interfering treatment.

## Reconsolidation of maladaptive memories in depression

The evidence that N_2_O can interfere with the (re)consolidation of maladaptive memories raises the intriguing possibility that this may be one mechanism through which N_2_O exerts its antidepressant effects. This hypothesis is yet to be tested directly in humans in the context of depression. However, there is some initial evidence from a preclinical model of affective learning in rodents that NMDAR antagonists may be able to impact negative memory biases, which are hypothesised to maintain depressive symptoms in humans [131]. By manipulating the timing of treatment relative to learning episodes, this preclinical model allows different phases of reward learning and memory to be targeted pharmacologically [131–133]. Studies using this paradigm have demonstrated potentially distinct mechanisms of action of conventional serotoninergic and rapid-acting glutamatergic antidepressants. Specifically, venlafaxine— a serotonin and norepinephrine reuptake inhibitor (SNRI)— did not affect established memories for emotional associations, but instead impacted new emotional learning [133]. In contrast, ketamine did not appear to impact new learning, but, rather, substantially affected memory for previously learned emotional associations as formerly trained negative biases were attenuated by ketamine treatment [132, 133]. Whilst it is important to note that this specific effect in rodents is yet to be tested using N_2_O, based on the close overlap with ketamine in terms of i) molecular pharmacology [29, 37, 134], ii) mechanisms of action at the cellular/circuit level [37], and iii) psychopharmacological effects in humans [40, 135], similar modulation of memory might be expected. Indeed, both ketamine and N_2_O have been reported to interfere with the reconsolidation of other maladaptive memories in humans [124, 125, 130].

It has been suggested that the divergent mechanisms highlighted by the rodent model may be linked with the differing speeds at which glutamate-based and conventional serotonergic antidepressant drugs elicit their therapeutic effects in humans. That is to say, in the case of SSRIs and SNRIs, mood improvements have been reported to emerge only after many weeks despite the observance of more rapid shifts (e.g., after a few hours) in new emotional learning [136, 137]. To explain this delay in symptom reduction, it has been posited that the translation of initial changes in affective learning to subsequent subjective mood improvements is dependent on interactions with the social environment. With these conventional, slower-acting antidepressants, effective treatment might therefore be conceptualised as the patient coming to view the (social) world through positively shifted lenses. Over time, evidence against depressive cognitive biases accumulates through the consolidation of new positive emotional associations, which eventually outweigh the pervasive maladaptive memories [138–141]. In contrast, however, the rapid action of glutamatergic antidepressants may, at least in part, be explained by the alteration of existing emotional memories, as opposed to the gradual accumulation of new learning. Whilst more specific study of these effects in humans is required, the potential for these novel treatments to target the reconsolidation of maladaptive memories in depression, such as those for schema-forming adverse life events, is intriguing.

## Open questions and future directions

There are a number of currently unanswered questions which should be addressed by future research. Firstly, it would be useful to explore whether the same patterns of affective bias modifications observed in animals following ketamine treatment are also seen for N_2_O. This could be done using a similar, if not identical, protocol to the rodent model of affective learning described earlier. Unveiling whether N_2_O influences new emotion-based learning, alters memory for previously established negative biases, or affects both— as has been reported for another rapid-acting antidepressant, psilocybin [132]— will be particularly pertinent to the understanding of this drug’s mechanism of action. Furthermore, uncovering whether N_2_O is able to shift affective biases associated with human autobiographical memories would also be useful. To aid this exploration, an experimental medicine model has been developed, which enables the impact of pharmacological treatments on established autobiographical memories to be assessed. This task is currently being used to evaluate the impact of both ketamine (NCT05809609) and N_2_O (NCT06557642) on the recollection of autobiographical memories. It is hypothesised that, through their interference with memory reconsolidation processes, these NMDAR antagonists could affect a number of parameters associated with the memory biases observed in depression. This ongoing work will also help elucidate whether ketamine and N_2_O might exert their rapid-acting antidepressant effects through parallel neuropsychological mechanisms.

It would also be particularly worthwhile to determine which (if any) pre-existing patient characteristics (e.g., genetic or endophenotypic individual variations [135]) and/or therapeutic contextual factors (setting) might moderate the effect of pharmacological agents on maladaptive memory expression. While targeting human memory reconsolidation may still offer a potentially transformative therapeutic strategy for a number of psychiatric conditions, the initial enthusiasm in this area has given way to a more realistic perspective. In particular, there is an increasing appreciation of the relevant technical complexities, such as the so-called boundary conditions that might limit the extent to which well-established memories can be destabilised, and the degree to which they can be ‘overwritten’ [112]. There are still many open questions and more recent research has shifted towards understanding the ‘permissive conditions’ for memory modification. These refer to the interactions between individual expectations formed by learning experience, the precision of these expectations, or ‘priors’, the characteristics of the memory reactivation (e.g., number of reminder cues, duration and timing of reactivation session) and the choice of experimental manipulation designed to induce a violation of expectation (and thus presumably prediction error) [142].

The need to establish optimised therapeutic procedures is further underlined by evidence that there may be conditions under which maladaptive memory expression is instead *enhanced* [143, 144]. Observational studies have suggested that ketamine administration shortly after trauma exposure may be associated with elevated acute and sustained post-traumatic stress symptomatology [145, 146]. Despite a number of limitations associated with these studies (e.g., lack of appropriate controls or small sample sizes), they highlight the need for caution when using memory therapeutic strategies that modulate NMDAR functioning in clinical populations [147].

It is also important to acknowledge that the molecular mechanisms through which N_2_O and ketamine exert their rapid antidepressant effects are currently unknown. Although previous work has focused on NMDARs, mu and/or kappa opioid receptor-mediated effects are also likely to be important to the antidepressant effects exerted by these pharmacological agents [148, 149]. Moreover, the opioid system is implicated in both affect and memory modulation [150]. N_2_O and ketamine (indirectly via its metabolites) also interact with other non-NMDA receptors [32, 42], which may contribute to their antidepressant profiles [151, 152], but this remains contentious (see [153, 154]). Overall, the multiple molecular targets highlighted here point to the complex mechanism that likely exists for the antidepressant action of dissociative anaesthetics.

Finally, from an implementation perspective, it must be acknowledged that N_2_O is a greenhouse gas, and high levels of ambient N_2_O in occupational settings can be hazardous to healthcare workers’ health [155]. In order to encourage large-scale adoption of N_2_O as a treatment for depression, it will therefore also be important to address environmental concerns and invest in gas destruction technologies, monitoring systems, and infrastructures that reduce the climate impact of N_2_O emissions (e.g., ‘cracking’) [156].

## Conclusion

In summary, the pharmacologically-mediated modification of negative memory biases in depression may offer a promising target for novel therapeutic agents. The rationale for considering N_2_O as a memory-based antidepressant treatment is based on i) its similar antidepressant profile to ketamine (i.e., rapid mood improvements via molecular pathways that may involve NMDA and non-NMDA glutamate receptors, as well as opioid receptor mediated effects), ii) the role of NMDARs in the molecular and neural underpinnings of memory reconsolidation, iii) the impact of N_2_O on human memory reconsolidation in other contexts, such as hazardous alcohol use, and iv) the action of other NMDAR antagonists and rapid-acting antidepressants in animal models of affective biases, learning, and memory. Whilst the last decade has seen tremendous progress in these areas, future research efforts should be directed to addressing a number of unanswered questions regarding N_2_O’s antidepressant effects. Further exploration of this topic could be particularly valuable given the attractive features that N_2_O appears to have compared with other glutamatergic (e.g., ketamine) and conventional serotonergic (e.g., SSRIs) treatment options for depression.
